# SC79 protects dopaminergic neurons from oxidative stress

**DOI:** 10.18632/oncotarget.23538

**Published:** 2017-12-20

**Authors:** Yan Xu, Ya-Wen Gao, Yu Yang

**Affiliations:** ^1^ Geriatrics Department, The Second Xiang Ya Hospital of Central South University, Changsha, China

**Keywords:** SC79, Akt, dopaminergic neurons, oxidative stress

## Abstract

Oxidative stress could lead to dopaminergic neuronal cell death. SC79 is a novel, selective and highly-efficient Akt activator. The current study tested its effect in dopaminergic neurons with oxidative stress. In both SH-SY5Y cells and primary murine dopaminergic neurons, pre-treatment with SC79 largely inhibited hydrogen peroxide (H_2_O_2_)-induced cell viability reduction, apoptosis and necrosis. SC79 activated Akt in the neuronal cells, which was required for its neuroprotection against H_2_O_2_. Inhibition of Akt activation (by MK-2206 or AT7867) or expression (by targeted short hairpin RNA) largely attenuated SC79-induced neuroprotection. Further, CRISPR-Cas9-mediated Akt1 knockout in SH-SY5Y cells abolished SC79-induced neuroprotective function against H2O2. Reversely, forced activation of Akt by the constitutively-active Akt1 mimicked SC79-induced anti-H_2_O_2_ activity. Together, we conclude that activation of Akt by SC79 protects dopaminergic neurons from H_2_O_2_.

## INTRODUCTION

Parkinson's disease (PD) is a chronic and progressive neurological disorder. It is the second most common neurodegeneration disease after Alzheimer's disease (AD) [1]. PD is mainly caused by the premature death of dopaminergic neurons in the midbrain [2–4].

Oxidative stress is important in mediating death of dopaminergic neurons in PD pathogenesis [5]. Studies have shown that inhibition of mitochondrial respiratory chain complexes can have devastating consequences, causing excessive radical oxidative species (ROS) production and oxidative stress. This will lead to intracellular calcium overload, lipid peroxidation, DNA damages, and excitotoxicity. Together, they ultimately induce neuronal cell death and apoptosis [5]. Mitochondrial respiratory chain complexes complex I inhibitors, *i.e.* 6-OHDA and 1-methyl-4-phenyl-1,2,3,4-tetrahydropyridine (MPTP), have been widely utilized to create PD animal and cellular models [5]. Further, hydrogen peroxide (H_2_O_2_) and other ROS were added directly to the cultured dopaminergic neurons to mimic oxidative stress injuries [6, 7].

Akt, also known as PKB, is a well-established pro-survival signaling [8–10]. Various stimuli, including insulin, growth factors, cytokines and cell stress, were shown to activate Akt, causing Akt phosphorylation at Ser-473 and Thr-308 [8]. Activated Akt then phosphorylates its substrates, including GSK3β, mTOR, BAD, CREB and the Forkhead transcription factors [8–10], which promote cell survival [8–10]. Activation of Akt was shown to efficiently protect neurons or neuronal cells from oxidative stress [11–13].

A recent study has characterized a novel small molecule compound SC79 as a selective, highly-efficient and cell-permeable Akt activator [14]. It has been previously shown that SC79 specifically and directly binds to the PH domain (pleckstrin homology domain) of Akt, inducing a conformational change which favors its activation [14]. At the molecular level, SC79 is shown to uniquely inhibit Akt membrane translocation, but activating Akt in the cytosol [14]. Studies have confirmed that SC79 could induce Akt phosphorylation at both Ser-473 and Thr-308 [15–17]. It has displayed profound cytoprotective function in different experimental settings [14–19]. For instance, SC79 was shown to inhibit excitotoxicity and to alleviate stroke-induced neuronal cell death [14]. SC79 administration *in vivo* could also alleviate early brain injuries [18]. Interestingly, a recent study however demonstrated that SC79 was in-effective to protect rat heart from ischemic injuries [20]. The current study tested the potential effect of this novel Akt activator in dopaminergic neurons when facing oxidative stresses.

## RESULTS

### SC79 protects dopaminergic neurons from H_2_O_2_

**Figure 1:**
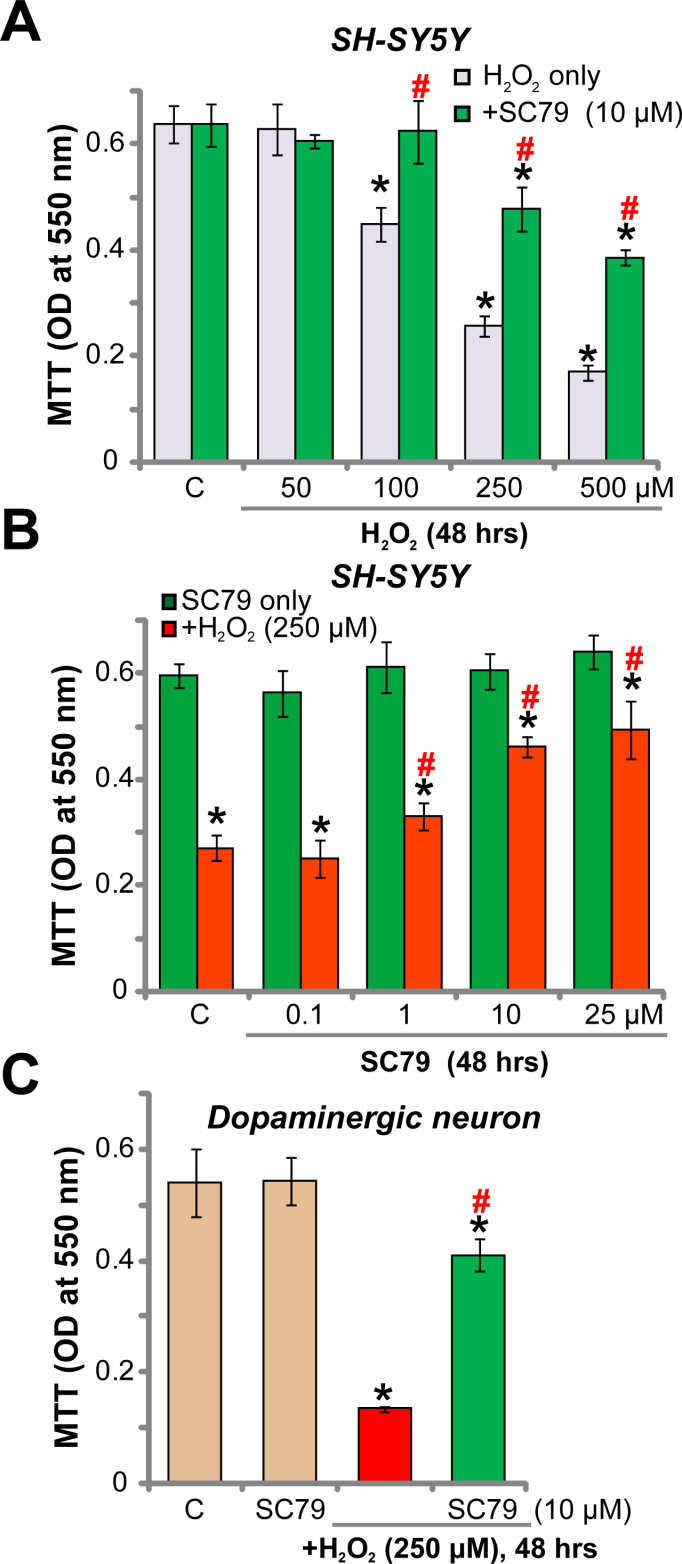
SC79 protects dopaminergic neurons from H_2_O_2_ SH-SY5Y cells (**A** and **B**) or the primary cultured murine dopaminergic neurons (**C**) were pre-treated for 30 min with SC79 (at tested concentration), followed by hydrogen peroxide (H_2_O_2_) stimulation, cells were further cultured in the medium for 48 hours, when MTT assay was performed to test cell survival (A–C). Bars indicate standard deviation (SD, *n* = 5). “C” stands for untreated control cells. ^*^p < 0.05 vs. “C” group. ^#^*p* < 0.05 vs. H_2_O_2_ only treatment (no SC79). Each experiment was repeated four times and similar results were obtained.

As discussed, Akt is a key pro-survival signaling [8, 21]. SC79 is newly-developed Akt activator [15–18, 20], we therefore wanted to know if SC79 could rescue dopaminergic neuron from hydrogen peroxide (H_2_O_2_). SH-SY5Y cells are well-established human dopaminergic neuronal cells [22–24]. MTT assay results in Figure 1A demonstrated that treatment with H_2_O_2_ in SH-SY5Y cells dose-dependently inhibited cell survival. Significantly, pre-treatment with SC79 (10 μM) largely attenuated H_2_O_2_-induced survival reduction in SH-SY5Y cells (Figure 1A). The concentration of SC79 (10 μM) was determined based on previous studies [15–17].

SC79's titration experiments were also performed. As shown in Figure 1B, treatment with SC79 alone at tested concentrations (from 0.1–25 μM) failed to change the viability of SH-SY5Y cells. Yet, SC79-mediated anti-H_2_O_2_ neuroprotection was dose-dependent (Figure 1B). It should be noted that a relative low concentration of SC79 (0.1 μM) failed to inhibit H_2_O_2_-induced anti-SH-SY5Y cell activity (Figure 1B). We also performed the above treatments in the primary cultured murine dopaminergic neurons. MTT assay results showed that adding H_2_O_2_ (250 μM) for 48 hours induced dramatic viability reduction in the primary dopaminergic neurons (Figure 1C). Such effect by H_2_O_2_ was significantly attenuated with pre-treatment of SC79 (10 μM) (Figure 1C). Together, these results show that SC79 protects dopaminergic neurons from H_2_O_2_.

### SC79 inhibits H_2_O_2_-induced apoptosis activation in dopaminergic neurons

**Figure 2:**
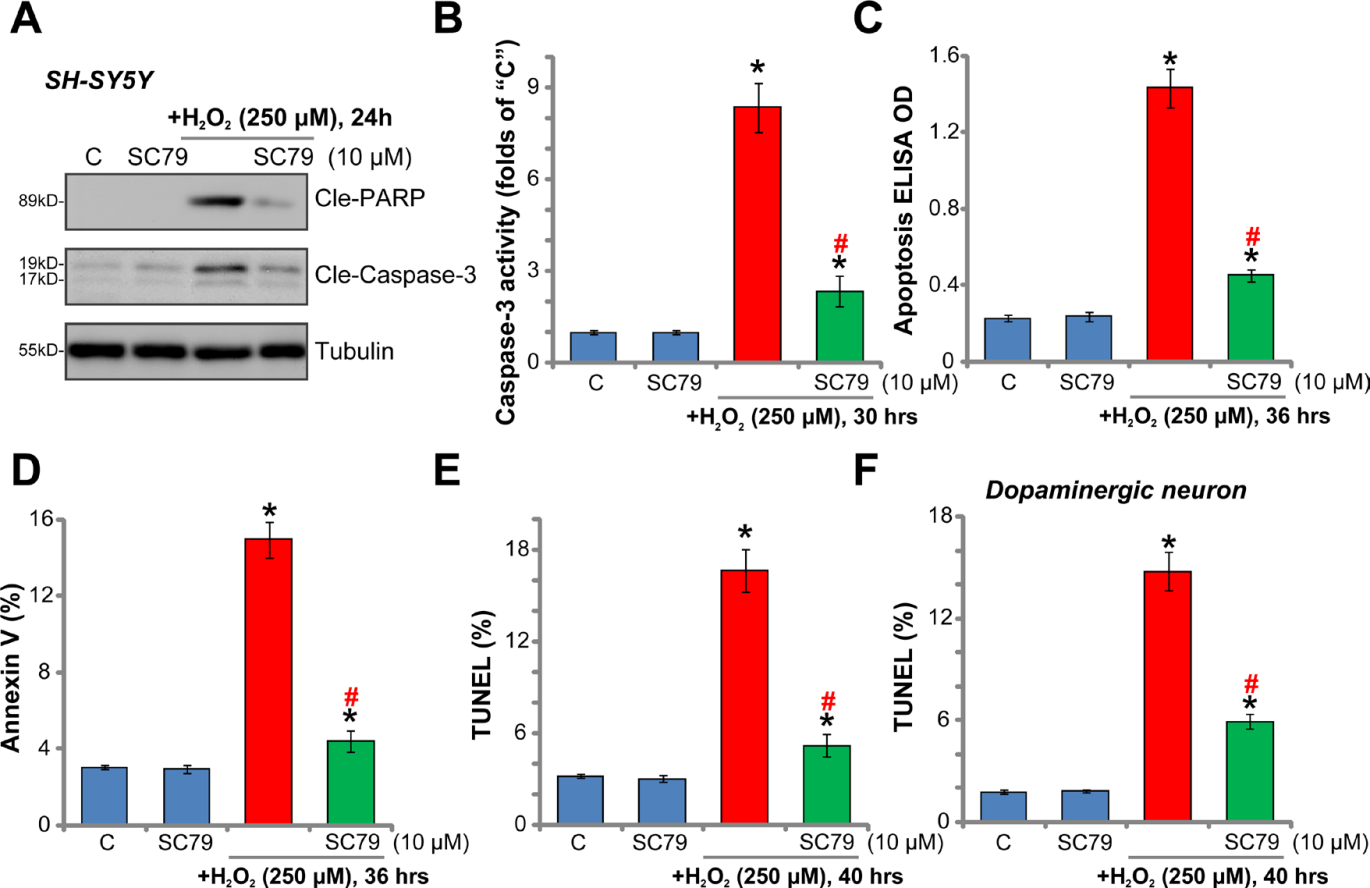
SC79 inhibits H_2_O_2_-induced apoptosis activation in dopaminergic neurons SH-SY5Y cells (**A**–**E**) or the primary cultured murine dopaminergic neurons (**F**) were pre-treated for 30 min with SC79 (10 μM), followed by hydrogen peroxide (H_2_O_2_, 250 μM) stimulation, cells were further cultured in the medium for the indicated time period, cell apoptosis was tested by the assays mentioned in the text (A–F). Bars indicate standard deviation (SD, *n* = 5). “C” stands for untreated control cells. ^*^p < 0.05 vs. “C” group. ^#^*p* < 0.05 vs. H_2_O_2_ only treatment (no SC79). Each experiment was repeated three times and similar results were obtained.

The potential effect of SC79 on H_2_O_2_-induced cell apoptosis was also tested. A panel of different apoptosis assays were applied. As demonstrated, H_2_O_2_ (250 μM) treatment in SH-SY5Y cells induced apoptotic cleavage of both poly (ADP-ribose) polymerase (PARP) and caspase-3 (Figure 2A), which was accompanied with significantly increased caspase-3 activity (Figure 2B) and Histone-bound DNA content (Figure 2C). Additionally, the percentage of SH-SY5Y cells with Annexin V-staining (Figure 2D) and TUNEL (dUTP nick-end labeling)-staining (Figure 2E) was also increased robustly with H_2_O_2_ (250 μM) treatment. These results clearly indicated that H_2_O_2_ activated apoptosis in SH-SY5Y neuronal cells. Remarkably, the results of these apoptosis assays all demonstrated that pre-treatment with SC79 (10 μM) dramatically inhibited H_2_O_2_-induced SH-SY5Y cell apoptosis (Figure 2A–2E).

In the primary murine dopaminergic neurons, SC79 similarly inhibited H_2_O_2_-induced apoptosis activation (TUNEL nuclei staining increase, Figure 2F). It should be noted that SC79 alone failed to induce neuronal cell apoptosis (Figure 2A–2F). Together, these results clearly show that SC79 inhibits H_2_O_2_-induced apoptosis activation in dopaminergic neurons.

### SC79 inhibits H_2_O_2_-induced programmed necrosis in dopaminergic neurons

**Figure 3:**
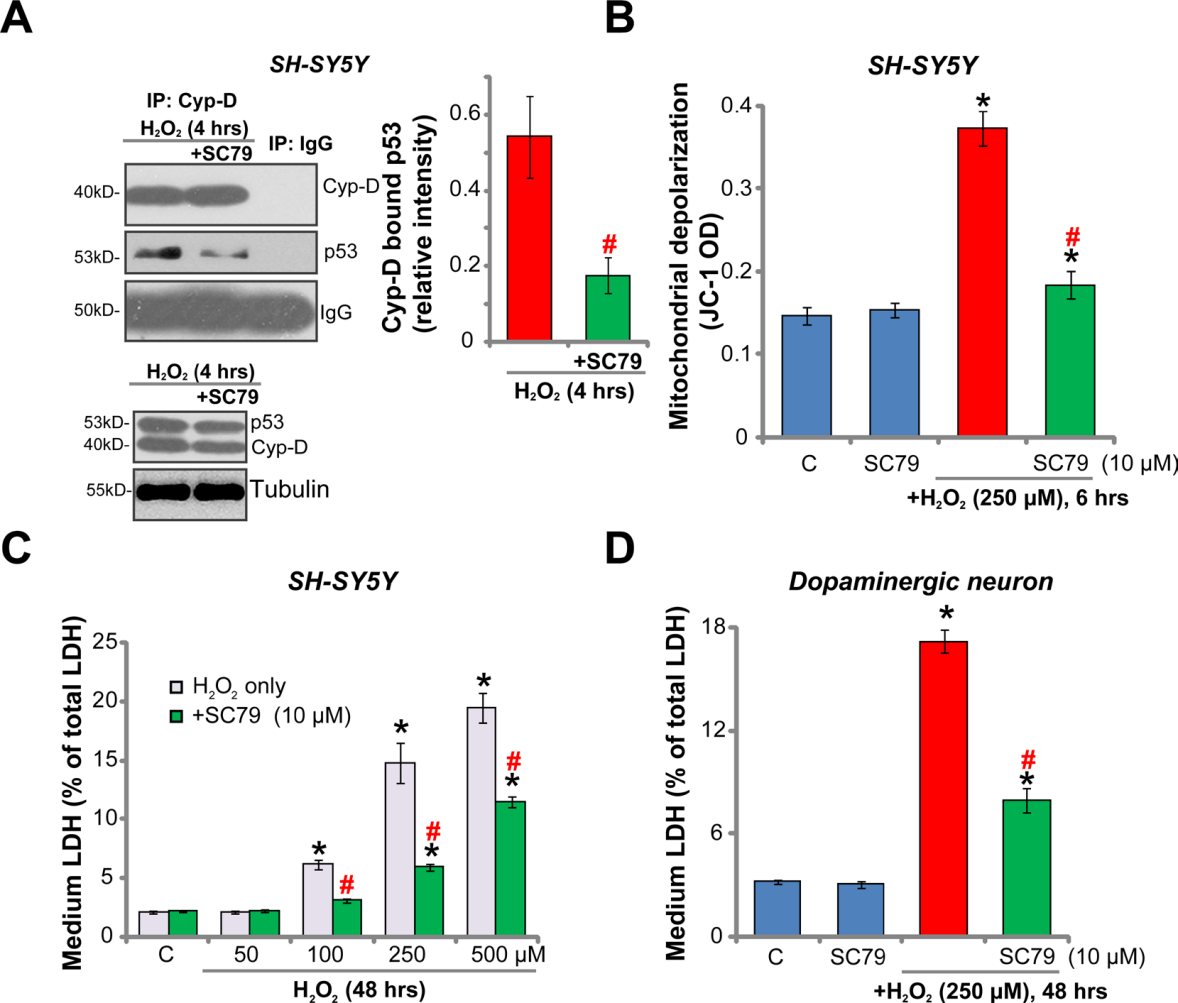
SC79 inhibits H_2_O_2_-induced programmed necrosis in dopaminergic neurons SH-SY5Y cells (**A**–**C**) or the primary cultured murine dopaminergic neurons (G) were pre-treated for 30 min with SC79 (10 μM), followed by hydrogen peroxide (H_2_O_2_, at indicated concentration) stimulation, cells were further cultured in the medium for the indicated time period, programmed necrosis was tested by p53-Cyp-D association (A, IP assay) and mitochondrial depolarization (JC-1 OD increase, B); LDH release in the conditional medium was also tested and its level was normalized to total LDH content (C and **D**). Bars indicate standard deviation (SD, *n* = 5). “C” stands for untreated control cells. ^*^p < 0.05 vs. “C” group. ^#^*p* < 0.05 vs. H_2_O_2_ only treatment (no SC79). Each experiment was repeated three times and similar results were obtained.

Recent studies have proposed that H_2_O_2_ and oxidative stress could also activate mitochondrion-dependent necrosis pathway [25–27]. This so-called “programmed necrosis” starts from p53 translocation to mitochondria, which forms a complex with the local mPTP (mitochondrial permeability transition pore) component protein cyclophilin-D (“Cyp-D”) [25–27]. The p53-Cyp-D association will lead to mitochondrial depolarization and cell necrosis [25–27]. The immunoprecipitation (“IP”) assay results in Figure 3A demonstrated that H_2_O_2_ (250 μM) treatment in SH-SY5Y neuronal cells induced p53-Cyp-D association, which was followed by mitochondrial depolarization (indicated by monomeric JC-1 fluorescence intensity increase [28, 29], Figure 3B). Remarkably, H2O2-induced p53-Cyp-D association and mitochondrial depolarization were significantly inhibited by SC79 pre-treatment (Figure 3A and 3B). p53 and Cyp-D expressions were unchanged by SC79 (Figure 3A, Input). Furthermore, H_2_O_2_ dose-dependently induced lactate dehydrogenase (LDH) medium release, which is the routine indicator of cell necrosis [30]. The pro-LDH release function by H_2_O_2_ was again largely inhibited by SC79 (Figure 3C). In the primary murine dopaminergic neurons, H_2_O_2_ (250 μM)-induced LDH release was also alleviated with pre-treatment of SC79 (10 μM) (Figure 3D). SC79 alone failed to induce cell necrosis (Figure 3A–3D). These results indicate that, besides apoptosis inhibition, SC79 also attenuates H_2_O_2_-induced programmed necrosis in dopaminergic neurons.

### Akt inhibition abolishes SC79-mediated neuroprotection against H_2_O_2_

**Figure 4:**
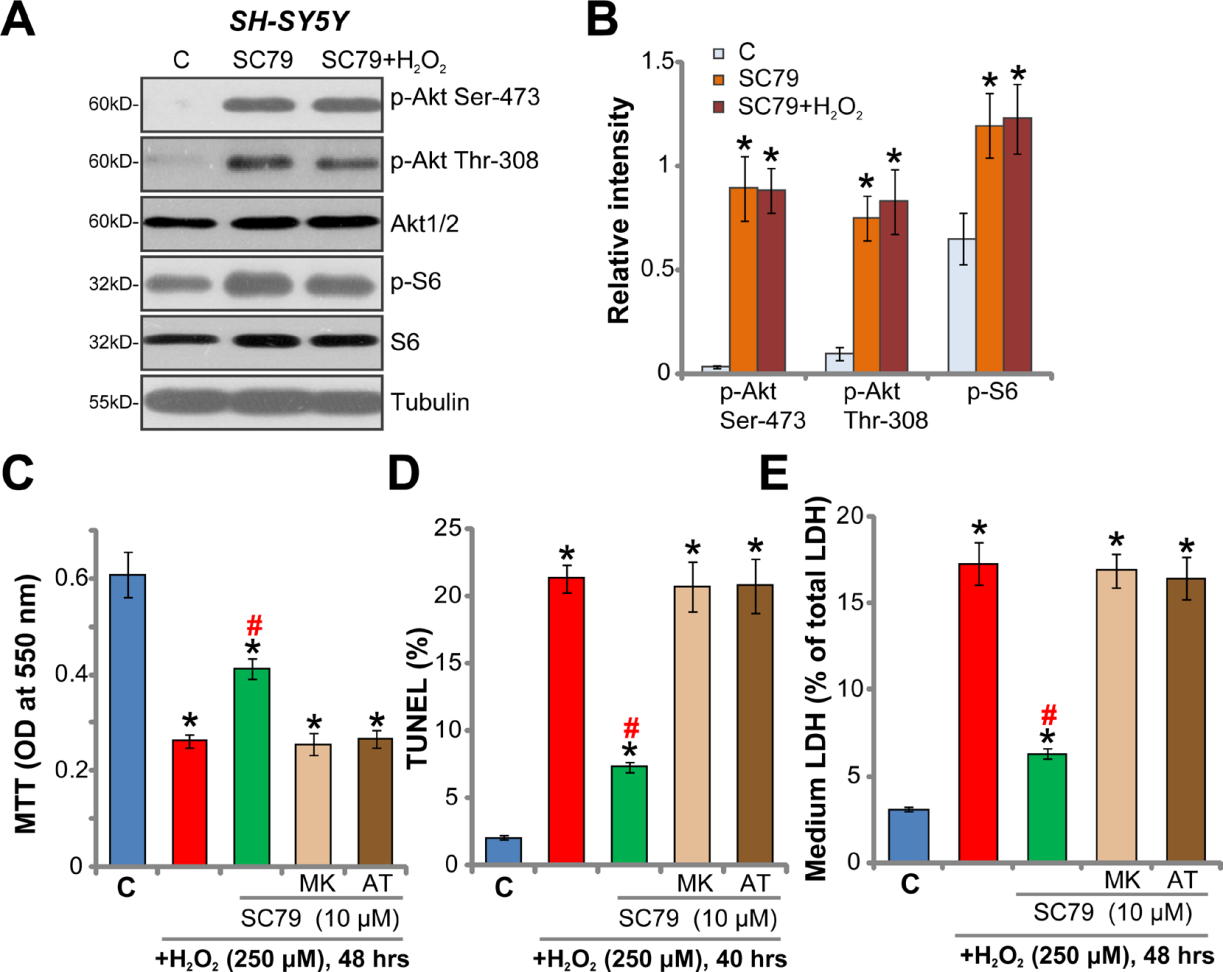
Akt inhibition abolishes SC79-mediated neuroprotection against H_2_O_2_ SH-SY5Y cells were treated with SC79 (10 μM) or plus hydrogen peroxide (H_2_O_2_, 250 μM) for 2 hours, total cell lysates were collected, and listed proteins were tested by the Western blotting assay (**A**). Protein phosphorylation (vs. the total protein) was quantified (**B**). SH-SY5Y cells were pre-treated for 30 min with SC79 (10 μM) or plus MK-2206 (“MK”, 5 μM)/ AT7867 (AT, 5 μM), followed by hydrogen peroxide (H_2_O_2_, 250 μM) stimulation, cells were further cultured in the medium, cell survival, apoptosis and necrosis were tested by MTT assay (**C**), TUNEL staining assay (**D**), and LDH release assay (**E**), respectively. Bars indicate standard deviation (SD, *n* = 5). “C” stands for untreated control cells. ^*^p < 0.05 vs. “C” group. ^#^*p* < 0.05 vs. H_2_O_2_ only treatment (no SC79). Each experiment was repeated three times and similar results were obtained.

SC79's effect on Akt signaling in neuronal cells was tested. The Western blotting assay results in Figure 4A demonstrated that SC79 (10 μM, 2 hours) potently increased phosphorylation of Akt at two key sites, Ser-473 and Thr-308 [8, 9], in SH-SY5Y cells. Meanwhile, phosphorylation of S6 (at Ser-235/236), the key Akt downstream protein, was also significantly boosted (Figure 4A). These results confirmed significant Akt activation by SC79 in SH-SY5Y cells. Notably, SC79-induced Akt activation was not inhibited by H_2_O_2_ (250 μM) treatment (Figure 4A, also see the quantified results in Figure 4B). As demonstrated in Supplementary Figure 1, treatment with SC79 for 30 min also induced significant Akt-S6 phosphorylation in SH-SY5Y cells. To study whether Akt activation was required for SC79-mediated neuroprotection, the specific Akt inhibitors were utilized, including MK-2206 [31, 32] and AT7867 [33, 34]. In the presence of the Akt inhibitors, SC79-mediated inhibitions on H_2_O_2_-induced viability reduction (MTT assay, Figure 4C), cell apoptosis (Figure 4D, TUNEL assay) and necrosis (Figure 4E, LDH release assay) were almost completely abolished (Figure 4C–4E). These results suggest that activation of Akt is indeed required for SC79-mediated neuroprotection against H_2_O_2_. Notably, both MK-2206 and AT7867 blocked Akt activation in SC79-treated SH-SY5Y cells (Data not shown).

### Activation of Akt mediates SC79-induced neuroprotection against H_2_O_2_

**Figure 5:**
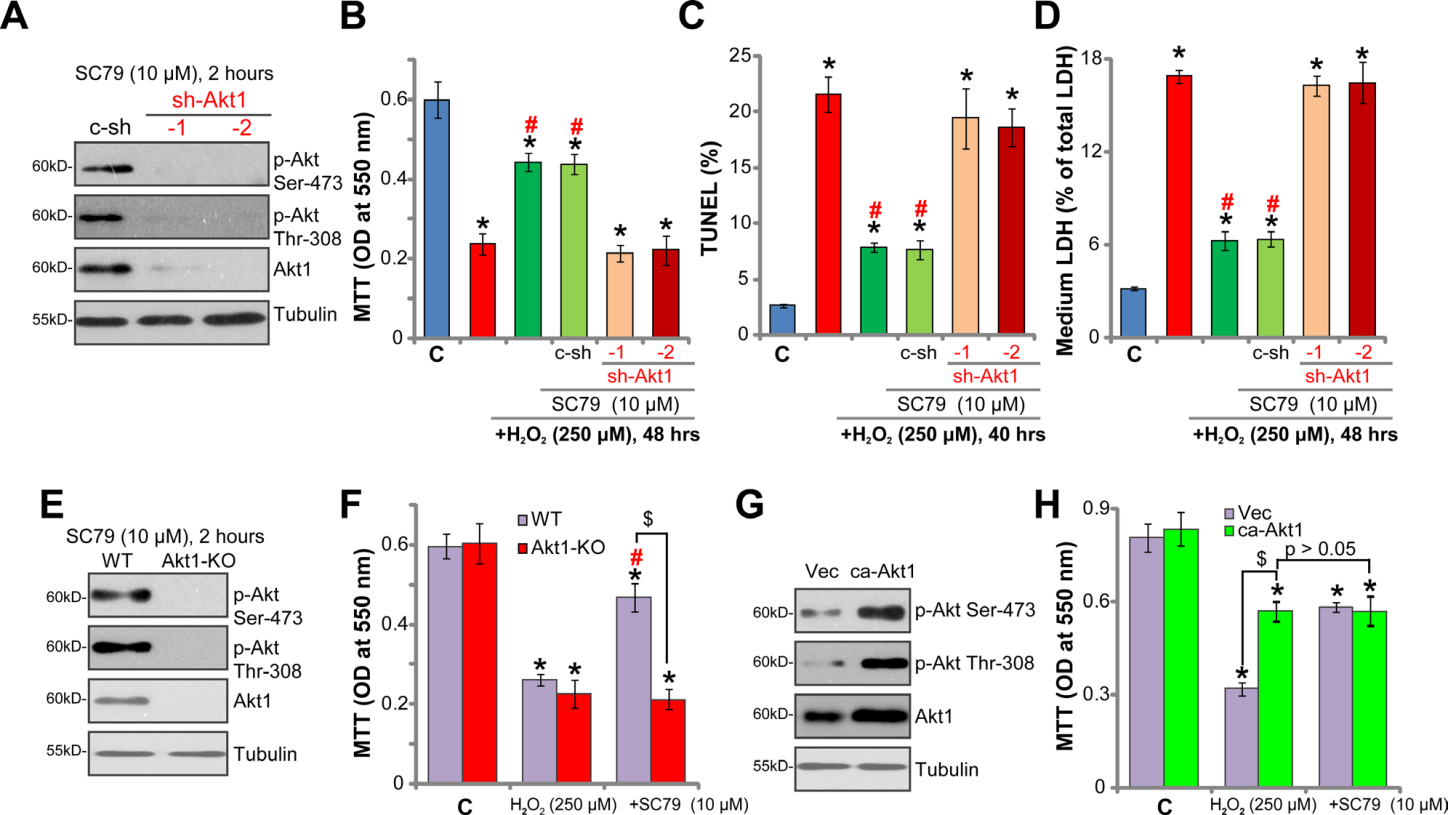
Activation of Akt mediates SC79-induced neuroprotection against H_2_O_2_ Stable SH-SY5Y cells, expressing Akt1 shRNA (“sh-Akt1”, Sequence “-1” or “-2”) or scramble control shRNA (“c-sh”) (**A**–**D**), the lenti-CRISPR-Cas9-Akt1 (“Akt1-KO”) or lenti-CRISPR-Cas9 control (“WT”) (**E** and **F**), as well as constitutively-active Akt1 (“ca-Akt1”) or the empty vector (“Vec”) (**G** and **H**), were treated with SC79 (10 μM, 30 min pre-treatment), or plus hydrogen peroxide (H_2_O_2_, 250 μM), Akt signalings were analyzed by Western blotting assay (A, E and G); Cell survival, apoptosis and necrosis were tested by MTT assay (B, F and H, 48 hours after H_2_O_2_ treatment), TUNEL staining assay (**C**), and LDH release assay (**D**), respectively. Bars indicate standard deviation (SD, *n* = 5). “C” stands for untreated control cells. ^*^p < 0.05 vs. “C” group. ^#^*p* < 0.05 vs. H_2_O_2_ only treatment (no SC79). ^$^*p* < 0.05 (F and H). Each experiment was repeated three times and similar results were obtained.

To exclude the possible off-target toxicities of the applied Akt inhibitors (MK-2206 and AT7867 [33]), genetic strategies were applied to inhibit Akt expression. A set of two different lentiviral short-hairpin RNAs (shRNAs, from Dr. Li [33]), targeting non-overlapping sequence of Akt1, were introduced to SH-SY5Y cells, and stable cells were established via puromycin selection (See Methods). Western blotting assay results in Figure 5A confirmed significant Akt1 downregulation by targeted shRNAs in the stable cells. SC79 (10 μM, 2 hours)-induced Akt phosphorylation (Ser-473 and Thr-308) was almost nullified in Akt1-shRNA-expressing cells (Figure 5A). Remarkably, SC79 was unable to rescue SH-SY5Y cells from H_2_O_2_ when Akt1 was silenced by the shRNAs (Figure 5B–5D). H_2_O_2_-induced viability reduction (Figure 5B), cell apoptosis (Figure 5C, TUNEL assay) and necrosis (Figure 5D, LDH release assay) were almost unchanged by SC79 in the Akt1-shRNA cells. The shRNA experimental results further indicate that Akt activation is required for SC79-mediated neuroprotection against H_2_O_2_.

To further support our hypothesis, CRISPR-Cas9 genome editing method was utilized to completely knockout Akt1 in SH-SY5Y cells. The lenti-CRISPR-Cas9-Akt1 construct was added to the SH-SY5Y cells, and stable cells were again established by puromycin selection. Western blotting assay results in Figure 5E confirmed Akt1 knockout in the stable cells, these cells were named as “Akt1-KO” cells. SC79 (10 μM, 2 hours)-induced Akt activation was obviously abolished in the Akt1-KO SH-SY5Y cells (Figure 5E). More importantly, SC79 was in-effective against H_2_O_2_ in the Akt1-KO cells (Figure 5F). Thus, Akt1 knockout also abolished SC79's neuroprotective actions. On the other hand, a constitutively-active Akt1 (“ca-Akt1”, also from Dr. Li [33]) was introduced to SH-SY5Y cells. The stable cells with ca-Akt1 showed an increased Akt expression and phosphorylation (Figure 5F). Cells with ca-Akt1 were also protected from H_2_O_2_, showing reduced viability reduction (Figure 5G). Notably, in the ca-Akt1-expressing SH-SY5Y cells, adding SC79 was unable to offer further protection against H_2_O_2_ (Figure 5H, *p* > 0.05). These results again confirm that activation of Akt mediates SC79-induced neuroprotection against H_2_O_2_.

## DISCUSSION

Recent studies have demonstrated the cytoprotective function of SC79. For example, Zheng *et al.*, showed that SC79 activated Akt signaling and protected myocardiocytes from oxygen and glucose deprivation (OGD)/re-oxygenation [15]. Li *et al.*, showed that activation of Akt by SC79 also protected osteoblasts from dexamethasone [16]. Further, Gong's group displayed that SC79-treated human eye retinal pigment epithelium cells were protected from UV radiation [17]. The same group found that SC79 could provoke Nrf2 signaling [17], which is a key anti-oxidant transcription factor [35]. In the current study, we found that SC79 induced profound Akt activation in the dopaminergic neuronal cells, which was evidenced by significant increase of Akt phosphorylation at both Ser-473 and Thr-308. Remarkably, pre-treatment with SC79 largely inhibited H_2_O_2_-induced neuronal cell viability reduction, cell apoptosis and necrosis. This could explain the superior neuroprotective function of this compound. It will be certainly interesting to test the activity of this compound *in vivo*.

Activation of Akt has proven to be a good strategy to protect neurons/neuronal cells from oxidative stress [11–13]. Our results provided strong evidences to show that Akt activation is required for SC79-mediated neuroprotection. Inhibition of Akt activation (by MK-2206 or AT7867) or expression (by targeted shRNAs) largely inhibited SC79-induced anti-H_2_O_2_ actions. Meanwhile, CRISPR-Cas9 genome editing-mediated complete Akt1 knockout also nullified SC79-mediated neuroprotection against H_2_O_2_. On the other hand, forced-activation of Akt by exogenous expression of ca-Akt1 mimicked SC79-mediated neuroprotection. More importantly, SC79 was unable to offer further protection against H_2_O_2_ in cells with ca-Akt1. These results clearly showed that activation of Akt is the primary mechanism of SC79-mediated neuroprotection against oxidative stresses.

It has been previously shown that total Akt- and phosphorylated-Akt-containing dopaminergic neurons were severely reduced in the brain in PD [36]. Interestingly, however, total Akt and phosphorylated-Akt were retained in degenerating dopaminergic neurons in areas of advanced PD pathology [36]. These results suggested that therapeutic agents activating Akt may have pro-neuronal survival capabilities in advanced PD [36]. Considering that previous studies have confirmed that SC79 could pass the blood brain barrier (BBB) [14], our results provide a theoretical basis for this compound to treat PD and possible other neurodegenerative diseases.

## METHODS

### Reagents

Hydrogen peroxide (H_2_O_2_) was purchased from Sigma (Shanghai, China). MK-2206, AT7867 and SC79 were provided by Selleck (Shanghai, China). All antibodies utilized in this study were obtained from Cell Signaling Tech (Danvers, MA).

### Culture of SH-SY5Y cells

The human dopaminergic neuroblastoma cell line, SH-SY5Y, was purchased from ATCC. SH-SY5Y cells were maintained at 37°C in 5% in CO_2_ in DMEM/F12 medium, supplemented with 5% fetal bovine serum (FBS) and penicillin/streptomycin. The cell culture reagents were purchased from Biyuntian (Suzhou, China).

### Culture of primary murine dopaminergic neurons

Murine dopaminergic neurons were cultured as described [37]. Briefly, tissues of the ventral mesencephalon containing dopaminergic neurons were dissected from murine embryo (C57/B6) on day-14 of gestation, which were subjected to trituration into single cell suspension. Cells were plated in mixed hormone MEM (MHM) supplemented with 1% FBS on poly-D-ornithine and fibronectin-coated glass slides (1 × 10^5^ cells/well). Forty-eight hours after initial plating, the medium was renewed and the cells were utilized for the further experiments.

### Cell viability assay

Cells were cultured in 96 well-tissue culture plate at 5 × 10^3^ cells per well. Following the applied treatment, the cell viability was tested via the MTT (3-(4, 5-dimethyl-2-thiazolyl)-2, 5-diphenyl-2H-tetrazolium bromide) using the attached protocol (Sigma, Shanghai, China). The MTT's optical density (OD) value (at 550 nm), dissolved in DMSO, was recorded.

### Western blotting assay and immunoprecipitation (IP) assay

Cells were cultured in 6 well-tissue culture plate at 2 × 10^5^ cells per well. Following the applied treatment, cells were lysed in the SDS-sample lysis buffer (pH 6.8) with 50 mM Tris-HCl, 2% SDS, 10% glycerol, 1 mM PMSF, 2 mM EDTA. Quantified proteins in total cell lysates were separated by the 10–12.5% sodium dodecyl sulfate-polyacrylamide gel electrophoresis (SDS-PAGE), and were transferred onto polyvinylidene difluoride membranes (Hybond-P, Amersham, Shanghai, China). The blots were then blocked in 5% milk dissolved in PBS containing 0.1% Tween 20 and indicated primary antibodies. The horseradish peroxidase (HRP)-conjugated secondary antibodies were then added to visualize the targeted protein bands using the ECL detection reagents (Amersham). For the immunoprecipitation (IP) assay, the quantified total cell lysates (800 μg per sample) were pre-cleared and incubated with anti-Cyp-D antibody [38, 39]. The Cyp-D complex was then captured by the protein G-Sepharose beads (Sigma). Cyp-D-p53 association was tested by Western blotting assay.

### Caspase-3 activity assay

Cells were cultured in 6 well-tissue culture plate at 2 × 10^5^ cells per well. Following the treatment, neuronal cells were lysed in buffer A (100 mM HEPES/KOH, pH7.5, 10% sucrose, 0.1% CHAPS, 10mM DTT, 1% TritonX-100, 1 mg/ml PMSF) and then centrifugation at 12,000 × g for 15 min. For each treatment, 10 μg lysate proteins were mixed with the caspase-3 substrate Ac-DEVD-MCA (100 μM, Biyuntian, Wuxi, China), and the caspase-3 activity was determined by the fluorescence spectrophotometer the excitation filter of 310 nm and emission filter of 460 nm.

### Annexin V FACS assay

Cells were cultured in 10-cm culture dish at 1 × 10^6^ cells per well. After treatment, collected cells were then incubated in Annexin V solution (10 μg/mL, Biyuntian, Wuxi, China) for 15 minutes. Immediately prior to reading on a FACS Calibur flow cytometer (BD, Nanjing, China), 10 μg/mL of propidium iodide (PI, Invitrogen) was added. For fluorescence-activated cell sorting (FACS) assay, Annexin V^+/+^/ PI^−/−^ cells were marked as early apoptosis cells [40, 41]. Annexin V^+/+^/ PI^+/+^ cells were marked as late apoptosis cells [40, 41]. Annexin V ratio was recorded.

### TUNEL assay

The detailed protocol of TUNEL staining assay was described in detail in other studies [42, 43]. Cells were placed on the tissue culture slide at 1 × 10^3^ cells per slide. After the indicated treatment, TUNEL positive apoptotic nuclei ratio was calculated, from at least 200 cells of 5 random views (1: 100).

### Histone DNA enzyme-linked immunosorbent assay (ELISA) assay of cell apoptosis

Cells were cultured in 6 well-tissue culture plate at 2 × 10^5^ cells per well. The Histone-DNA ELISA Detection Kit (Roche, Palo Alto, CA) was applied to quantify cell apoptosis, which is based on the detection of apoptosis-associated Histone-bound DNA content. The ELISA OD at 405 nm was recorded.

### LDH assay of cell necrosis

LDH release to the conditional medium is commonly tested as a marker of cell necrosis. Cells were cultured in 6 well-tissue culture plate at 2 × 10^5^ cells per well. After the applied treatment, LDH level was tested via the commercial available two-step LDH detection kit (Promega, Shanghai, China). Medium LDH content was always normalized to the total LDH.

### Detection of mitochondrial depolarization (ΔΨm)

As described [44], mitochondrial membrane potential (MMP) reduction implies mitochondrial depolarization, which was tested by JC-1 fluorescent dye (Invitrogen, Shanghai, China) assay. With mitochondrial depolarization, monomeric JC-1 will be formed in the cytosol, exhibiting green fluorescence. Cells were cultured in 96 well-tissue culture plate at 5 × 10^3^ cells per well. After treatment, cells were stained with JC-1 (10 μg/mL) for 15 min under the dark. JC-1 intensity was tested by a fluorescence spectrophotometer with the excitation filter of 485 nm and emission filter of 527 nm.

### Akt1 shRNA knockdown

The two lentiviral Akt1 shRNAs (“−1/−2”), with non-overlapping sequences, as well as the scramble control shRNA, were provided by Dr. Xiangcheng Li [33]. Cells were cultured in 6 well-tissue culture plate at 1 × 10^5^ cells per well. The shRNA lentivirus (10 μL/mL medium per well) was added to cultured neuronal cells for 24 hours. Puromycin (2.0 μg/mL) was then included to select stable colonies for 6 days. Expression of Akt1 in the stable cells was always tested.

### Constitutively-activate Akt1 expression

The constitutively-activate Akt1 (ca-Akt1 [45]) and the empty vector were again provided by Dr. Xiangcheng Li [33]. Cells were cultured in 6 well-tissue culture plate at 1 × 10^5^ cells per well. The ca-Akt1 construct or the empty vector was transfected to the neuronal cells by Lipofectamine 2000 (Invitrogen, Shanghai, China). Stable cells were selected by puromycin (2.0 μg/mL) for 6 days. Expression of Akt1 in the stable cells was always tested.

### CRISPR/Cas9-mediated Akt1 knockout

The small guide RNA (sgRNA) targeting human Akt1 was chosen from the website (http://crispr.mit.edu/), and it was inserted into the lenti-CRISPR plasmid (Addgene). Cells were cultured in 6 well-tissue culture plate at 1 × 10^5^ cells per well. The CRISPR/Cas9 Akt1 construct was then transfected to SH-SY5Y cells. Stable cells were again selected by puromycin (2.0 μg/mL) for 6 days. Akt1 knockout in the stable cells was verified by Western blotting assay.

### Statistics analysis

The data were expressed as the mean ± standard deviation (SD). Data were first analyzed using one-way factorial analysis of variance (ANOVA). Student's *t*-test or Turkey's test was then performed to compare treated samples, and *p* < 0.05 was considered significant.

## SUPPLEMENTARY MATERIALS FIGURE


