# Supplementary material for: SC79 protects dopaminergic neurons from oxidative stress
Source: Oncotarget. 2017 Dec 20;9(16):12639–48. doi: 10.18632/oncotarget.23538 (PMC5849161; doi:10.18632/oncotarget.23538)
Supplement: Supplementary file 1 [file oncotarget-09-12639-s001.pdf]

SC79 protects dopaminergic neurons from oxidative stress

SUPPLEMENTARY MATERIALS

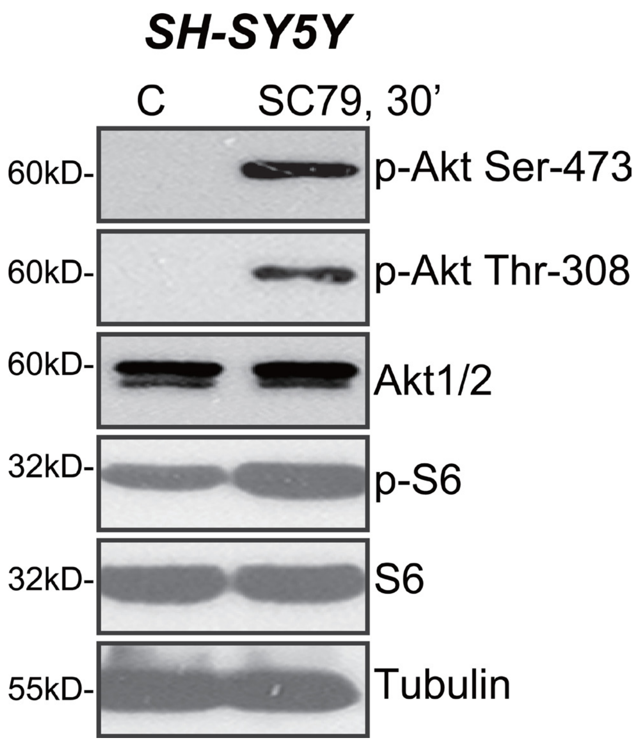

Supplementary Figure 1: SH-SY5Y cells were treated with SC79 (10 µM) for 30 min, total cell lysates were collected, and listed proteins were tested by the Western blotting assay. The experiment was repeated three times and similar results were obtained.
